# Factors Associated With Choriocarcinoma Syndrome Development in Poor-Risk Patients With Germ Cell Tumors

**DOI:** 10.3389/fonc.2022.911879

**Published:** 2022-06-17

**Authors:** Katarina Rejlekova, Katarina Kalavska, Marek Makovnik, Nikola Hapakova, Michal Chovanec, Valentina De Angelis, Jana Obertova, Patrik Palacka, Zuzana Sycova-Mila, Jozef Mardiak, Michal Mego

**Affiliations:** ^1^ 2^nd^ Department of Oncology, Faculty of Medicine, Comenius University and National Cancer Institute, Bratislava, Slovakia; ^2^ Oncology Department, National Cancer Institute, Bratislava, Slovakia; ^3^ Translational Research Unit, Faculty of Medicine, Comenius University, National Cancer Institute, Bratislava, Slovakia; ^4^ Radiology Department, National Cancer Institute, Bratislava, Slovakia

**Keywords:** choriocarcinoma syndrome, acute respiratory distress syndrome, germ cell tumors, poor-risk, induction chemotherapy, human choriogonadotropin

## Abstract

**Background:**

Germ cell tumors (GCTs) represent a highly curable cancer. However, a small proportion of poor-risk patients can develop choriocarcinoma syndrome (CS) connected with acute respiratory distress syndrome (ARDS) with a high mortality rate. Our retrospective study aimed to determine the risk factors of poor-risk GCTs susceptible to CS development.

**Patients and Methods:**

Using a computerized database and a systematic chart review, we identified the records of 532 patients with GCTs treated at the National Cancer Institute from 2000 to 2018. Ninety eligible patients with poor-risk GCTs based on IGCCCG classification were identified. All patients were treated with platinum-based induction chemotherapy. Clinicopathological variables were collected and analyzed in correlation with CS development.

**Results:**

Nine (10%) of 90 patients developed CS in a median of 1 day (1–9 days) after chemotherapy administration. All patients died shortly after the chemotherapy start with a median of 4 days (3–35 days) due to ARDS development. In univariate analysis, metastatic lung involvement ≥50% of lung parenchyma, choriocarcinoma elements in histology specimen, dyspnea, cough, hemoptysis, ECOG PS ≥2, weight loss, hemoglobin ≤100 g/l, and NLR ≥3.3 at the time of presentation were associated with CS development. In multivariate analysis, ECOG PS ≥2 and metastatic lung involvement ≥50% were independently associated with CS. All patients with these two characteristics developed CS, compared to 0% with zero or one of these factors (p < 0.000001).

**Conclusions:**

In our study, we identified factors associated with CS development. These factors might improve the risk stratification of the patients susceptible to CS and improve their outcome.

## Introduction

Despite the rarity of germ cell tumors (GCTs), they represent the most common solid tumor in men between the ages 15 and 39 years, associated with a high curability rate (1). However, a small portion of patients can develop choriocarcinoma syndrome (CS) connected with acute respiratory distress syndrome (ARDS) with a high mortality rate.

Moran-Ribon et al. defined a clinical entity, so-called super-high-risk GCTs, a subset characterized by respiratory distress, which was the major cause of death very early in the course of their treatment (2). The initial presentation characteristics of super-high-risk patients included three aspects: (1) two or more of the following symptoms: dyspnea, chest pain, hemoptysis, and weight loss, (2) far advanced disease: bulky pulmonary disease and a very high hCG level, and (3) presence of hypoxemia. The authors proposed a multifactorial etiology of acute pulmonary failure in those patients, and the clinical presentation was compatible with choriocarcinoma syndrome (CS), described by Logothetis et al. This syndrome is defined by hemorrhage from metastatic sites in patients with advanced GCTs with a high volume of choriocarcinoma elements, especially those with a hCG level over 50,000 IU/l (3). However, the exact understanding of CS development remains to be elucidated (4). The data describing ARDS development in poor-risk patients are limited and inconsistent. The authors hypothesized a possible aggravation of acute pulmonary failure by chemotherapy-induced/-associated inflammatory processes and local tumor lysis, but the degree to which chemotherapy is actually responsible for triggering the respiratory worsening remains unclear (5). It has been postulated that the underlying mechanism of this syndrome is massive cell death due to chemotherapy and consequent release of cytokines, potentially aggravated by alveolar hemorrhage (2, 5). Co-temporary infection may be an associated cause of death as well (5). The pronounced risk factors are extensive pulmonary metastases, hypoxemia, and, commonly, a very high level of hCG at the time of their presentation (2, 5, 6).

To date, there are only a few case reports and small retrospective series recording the ARDS development in patients with advanced GCTs trying to find the optimal therapeutic approach for them (2, 5–14). Massard et al. suggested in patients with extensive lung metastases and dyspnea and/or hypoxemia a modified induction regimen containing etoposide and cisplatin (EP) on days 1–3 with continuing full-dose chemotherapy after patients’ stabilization, which decrease the rate of ARDS development from 87% to 30%, with improvement of overall survival from 27% to 40% compared to initial full-dose chemotherapy (6). This approach is suggested by the European Germ Cell Cancer Consensus Group (EGCCCG) in patients with widespread lung metastases, pure choriocarcinoma, and a high choriogonadotropin level (15, 16). National Comprehensive Cancer Network (NCCN) guidelines recommend treatment of those patients in high-volume reference centers with optimalization of their chances for survival (17).

Until now, in the literature, we are missing an exact definition of the super-high-risk patients susceptible for CS and consecutive ARDS development as well as generally established risk factors. The aim of our study was to determine the factors associated with choriocarcinoma syndrome development in poor-risk GCTs patients treated in a tertiary cancer center.

## Patients and Methods

### Patients

**Figure 1:**
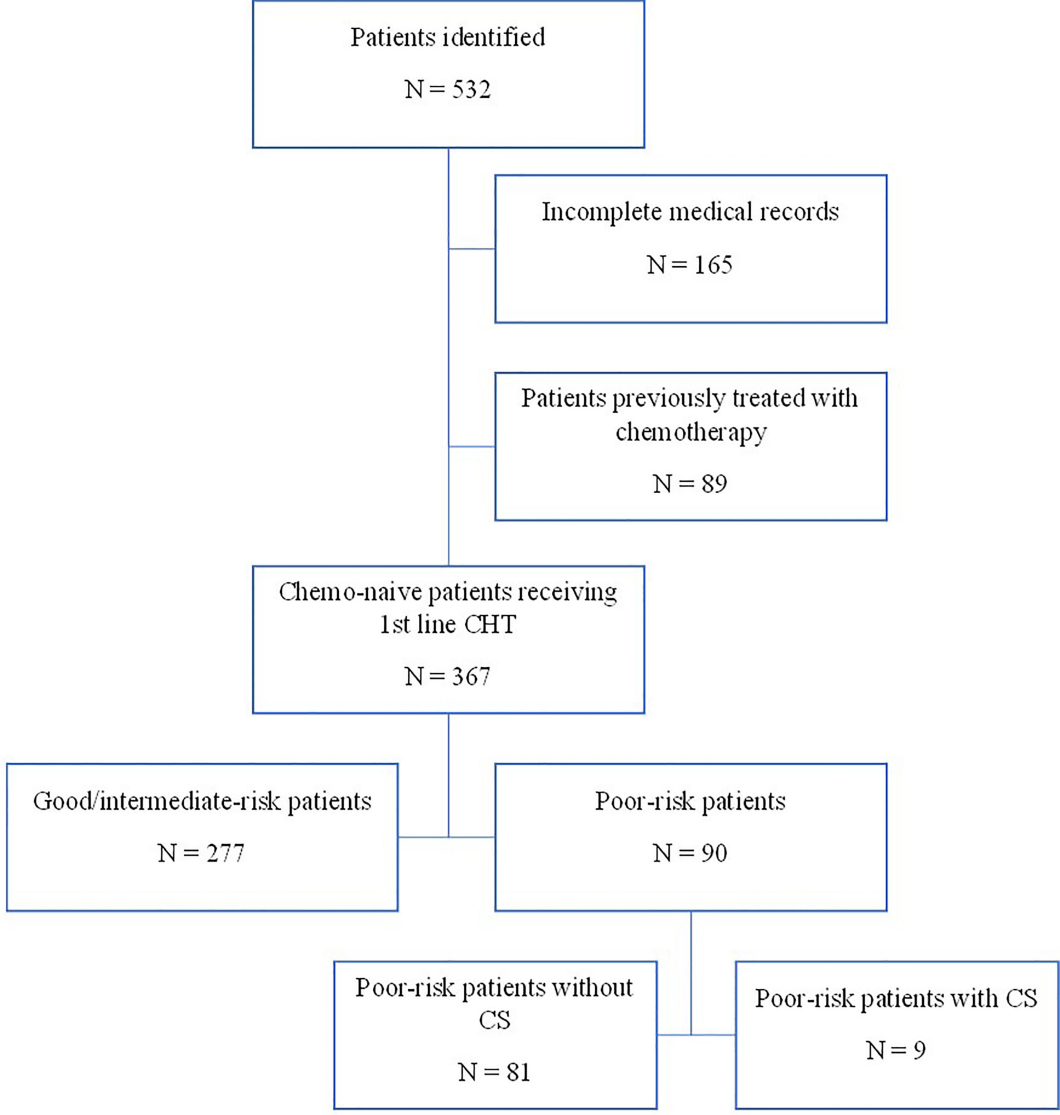
Flowchart of patients with GCTs treated in NCI.

Using a computerized database and a systematic chart review, we identified the records of 532 patients with GCTs treated with first-line chemotherapy at the National Cancer Institute in Slovakia from 2000 to 2018. We identified 90 eligible patients with poor-risk GCTs according to the IGCCCG classification (18–20) (**Figure 1**).

Eligible patients were ≥18 years of age with a chemo-näive GCT of any primary tumor site. When the primary tumor was in the testis, an orchiectomy was initially carried out in most of the cases and the diagnosis was confirmed by histological analysis according to the World Health Organization classification of GCTs (1). In cases requiring early chemotherapy treatment, the diagnosis of advanced GCTs was made without pathological evidence. In exceptional cases, this could involve a patient presenting with a typical picture of metastatic disease and highly elevated levels of tumor markers AFP) and/or hCG (21).

Data, including Eastern Cooperative Oncology Group (ECOG) performance status, level of hCG, metastatic lung involvement, dyspnea, hemoptysis, weight loss, pain grade ≥3, extent of retroperitoneal (RP) lymphadenopathy (LAP), presence of brain, liver metastases, and laboratory findings (level of hemoglobin, leucocytes/neutrophils, thrombocytes, fibrinogen, thyroid-stimulating hormone (TSH), free thyroxine (fT4), renal parameters, liver enzymes, LDH) at the time of presentation, were collected. Other data included the use of growth factors, incidence of febrile neutropenia, septic shock, need of intensive care unit (ICU) transfer, need of mechanical ventilation, paO_2_ at the time of ICU admission, and treatment-related death in a short period of time after the chemotherapy administration.

The study protocol was reviewed and approved by the Ethical Committee of the National Cancer Institute of Bratislava, Slovakia, and waiver for informed consent was granted.

### Definitions

*Choriocarcinoma syndrome* (*CS*) was defined as a life-threatening complication in poor-risk patients with GCTs, consisting of hemorrhagic manifestations of metastases (bleeding grade ≥3) within a few hours/days after the chemotherapy administration, followed by superinfection and/or sepsis, ending in life-threatening complications, septic shock with the need of circulation support, and/or ARDS with the need of mechanical ventilation.

The *systemic immune-inflammation index* (*SII*) is an index based on platelet (*P*), neutrophil (*N*), and lymphocyte (*L*) counts. It was calculated using the formula, SII = *P* × *N*/*L*, as defined previously. We stratified the patients into high and low SII using different cutoffs based on previous studies (22, 23) including the median value of our cohort as well.

The *neutrophil-to-lymphocyte ratio* (*NLR*) was obtained by dividing the absolute neutrophil count (cells/mm^3^) by the absolute lymphocyte count (cells/mm^3^). We stratified the patients into high (≥3.3) and low (<3.3) groups as described previously (22).

The *platelet-to-lymphocyte ratio* (*PLR*) was calculated as the ratio of absolute platelet count (cells/mm^3^) to absolute lymphocyte count (cells/mm^3^). We stratified the patients into high (≥170) and low (<170) groups as described previously (22).

### Digital Volumetry

Since the classic X-ray image is two-dimensional, it is not possible to measure volumetry as such. Therefore, we chose the possibility of measuring the density area of metastasis and lung parenchyma. which gave us the percentage of lung area affected by metastases, from the obtained two-dimensional X-ray image of the lungs in patients with GCTs (24). The next step was to categorize patients. We created four categories: i) category I included patients without metastatic disease of the lung; ii) category II included patients who had less than 30% of the lung parenchyma area affected; iii) category IIII included patients who had 30% to 49% of the lung area affected; and iv) category IV included patients who had of 50% or more of lung parenchyma affected.

Digital volumetry was undertaken in 67 (74%) poor-risk patients with initial X-rays available for evaluation. We could not perform digital volumetry from CT (in our center, we have a CT digital system for storage from 2008). The data on digitally stored CT scans due to the low number of patients would not be sufficient for our evaluation.

### Evaluation

Baseline examination included a complete physical examination, electrocardiogram, complete blood cell count (CBC), complete biochemistry, measurement of serum tumor markers (LDH, AFP, hCG), biochemistry, CT scan of the chest, abdomen, pelvis, testicular ultrasound, and MRI of the brain.

### Toxicity

Toxicity evaluation was performed according to CTC AE v.5.0. Life-threatening toxicity was defined as grade 4 except for hematological laboratory abnormalities (25).

### Statistical Analysis

Patient characteristics were tabulated. The characteristics were summarized using the mean or median (range) for continuous variables and the frequency (percentage) for categorical variables.

Normality of distribution was tested by the Kolmogorov–Smirnov test. For continuous variables, if data were normally distributed, sample means were tested by Student t-test or analysis of variance. For non-normally distributed data, the non-parametric Mann–Whitney U test or Kruskal–Wallis H test was used. For categorical variables, the chi-squared or Fisher’s exact test was performed.

Subsequently, multivariate logistic regression analysis included the variables significantly associated with CS development and clinicopathological factors identified in univariate analysis. Variables with *p* value ≤ 0.05 on univariate analysis were included in the multivariate analysis for CS development.

Follow-up was calculated from the date of chemotherapy start to the date of death or last follow-up. Progression-free (PFS) survival was calculated from the date of chemotherapy start to the date of progression of disease or death of any cause. Overall survival (OS) was calculated from the date of chemotherapy start to the date of death or last follow-up. PFS and OS were estimated using the Kaplan–Meier product-limit method. Statistical analyses were performed using NCSS 2007 software (26, Kaysville, UT, USA).

## Results

### Patients’ Characteristics

From November 2000 to May 2018, 90 consecutive patients with poor-risk features according to IGGGCCC were treated in our center. The baseline characteristics of the patients are summarized in **Table 1**. All poor-risk patients received platinum-based combinations for the first cycle of chemotherapy, whereas one patient received carboplatin monotherapy due to concern of volume overloading together with impaired renal functions (**Table 1**). Majority of patients received bleomycin, etoposide, and cisplatin combination (BEP); other utilized regimens included paclitaxel plus BEP (T-BEP, within the investigator-running clinical phase II trial in our center) (27, 28) or etoposide, ifosfamide, and cisplatin (VIP) (in case of bleomycin contraindication). Twenty-three (25%) patients received a reduced-dose induction regimen before planned full-dose chemotherapy based on expert-opinion decision in our department. The most often used modified induction regimen consisted of etoposide 100 mg/m^2^/day and cisplatin 20 mg/m^2^/day on days 1–3.

**Table 1:** Patient characteristics at the time of presentation.

Variable	N	%
**All**	90	100.0
**Age [years]**		
Median	28.5	
Range	17–63	
**Age [years]**		
<30	52	57.8%
<30–49>	20	22.2%
<40–49>	14	15.6%
≥50	4	4.4%
**ECOG**		
0	33	36.7%
1	28	31.1%
2	18	20.0%
3	10	11.1%
4	1	1.1%
**Primary tumor site**		
Testis	70	77.8%
Extragonadal	20	22.2%
Retroperitoneal	11	12.2%
Mediastinal	8	8.9%
Gl. pinealis	1	1.1%
**Histology**		
Pure choriocarcinoma	9	10.0%
Predominant choriocarcinoma	10	11.1%
Non-histologized	16	17.8%
EC	7	7.8%
YST	6	6.7%
Other mixed NSGCTs	42	46.7%
**Metastatic lung involvement**	*N=67*	
<30%	10	14.9%
<30–50>%	9	13.4%
>50%	16	23.9%
No MTS	32	47.8%
**CNS metastases**		
Yes	10	11.1%
No	80	88.9%
**Liver metastases**		
Yes	51	56.7%
No	39	43.3%
**hCG [mUI/mL]**		
<50,000	46	51.1%
≥50,000	44	48.9%
**AFP [μg/L]**		
<10,000	66	73.3%
≥10,000	24	26.7%
**Hemoglobin [g/L]**		0.0%
<100	24	26.7%
≥100	66	73.3%
**Dyspnea at rest**		
Yes	24	26.7%
No	66	73.3%
**Cough**		
Yes	30	33.3%
No	60	66.7%
**Chest pain**		
Yes	11	12.2%
No	79	87.8%
**Hemoptysis**		
Yes	14	15.6%
No	76	84.4%
**Weight loss ≥ 10%**		
Yes	38	42.2%
No	52	57.8%
**Pain grade ≥ 3**		
Yes	22	24.4%
No	68	75.6%
**Platelets [g/L]**		
≤300	34	37.8%
>300	56	62.2%
**Leukocytes [g/L]**	0	0.0%
≤10,000	28	31.1%
>10,000	62	68.9%
**CHT dose**		
Full	63	70.0%
Reduction	27	30.0%
**Induction CHT regimen**		
CBDCA	1	1.1%
CBDCA+VP-16	1	1.1%
EP	25	27.8%
BEP/T-BEP/VIP	63	70.0%
**DVT before CHT**		
Yes	12	13.3%
No	78	86.7%
**Primary prophylaxis of DVT**		
Yes	36	40.0%
No	54	60.0%
**Primary prophylaxis of FN**		
With (pegfilgrastim/filgrastim)	45	50.0%
Without	45	50.0%

EC, embryonal carcinoma; YST, yolk sac tumor; NSGCT, non-seminomatous germ cell tumor; hCG, choriogonadotropin; AFP, alphafetoprotein; CHT, chemotherapy; DVT, deep venous thrombosis; CHT, chemotherapy; FN, febrile neutropenia; DVT, deep venous thrombosis; CBDCA, carboplatin; VP-16, etoposide; EP, etoposide cisplatin; BEP, bleomycin etoposide cisplatin; T-BEP, paclitaxel-BEP; VIP, etoposide ifosfamide cisplatin.

### Characterization of Patients With CS Development

Nine (10%) of 90 poor-risk patients developed CS in a median of 1 day (range: 1–9 days) after the chemotherapy administration. The median age of these patients was 28 years (range: 21–58 years), and the median ECOG was 3 (range: 2–4). Seven patients had a testicular origin, while two patients had extragonadal primary origin (**Table 2**). Histological findings were available in four patients, while five patients were, due to the need of a prompt chemotherapy start, treated without pathological diagnosis. All patients had ≥50% of metastatic lung involvement. Patients with CS development had mean 88.2% lung involvement compared to 19.3% in patients without CS development (p < 0.0001).

**Table 2:** Univariate analysis of factors associated with CS development.

Variable	Number of pts	CS yes		CS no		p-value
	N	N	%	N	%	Pearson’s chi-square
**All**	90	9	9.6	81	90.4	
**Age [years]**						0.15
<30	52	5	9.6	47	90.4	
<30–49>	20	0	0.0	20	100.0	
<40–49>	14	3	21.4	11	78.6	
≥50	4	1	25.0	3	75.0	
**ECOG**						**<0.001**
0	33	0	0.0	33	100.0	
1	28	0	0.0	28	100.0	
2	18	2	11.1	16	88.9	
3	10	6	60.0	4	40.0	
4	1	1	100.0	0	0.0	
**Primary tumor site**						0.98125
Testis	70	7	10.0	63	90.0	
Extragonadal	20	2	10.0	18	90.0	
*Retroperitoneal*	*11*	*1*	9.1	10	90.9	
*Mediastinal*	*8*	*1*	12.5	7	87.5	
*Gl. pinealis*	1	0	0.0	1	100.0	
**Histology**						**0.0015**
Pure choriocarcinoma	9	3	33.3	6	66.7	
Predominant choriocarcinoma	10	1	10.0	9	90.0	
Non-histologized	16	5	31.3	11	68.8	
EC	7	0	0.0	7	100.0	
YST	6	0	0.0	6	100.0	
Other mixed NSGCTs	42	0	0.0	42	100.0	
**Metastatic lung involvement**	*67*	***9***	*13.4*	*58*	*86.6*	**0.00002**
<30%	10	0	0.0	10	100.0	
<30–50>%	9	0	0.0	9	100.0	
>50%	16	9	43.8	9	43.8	
No MTS	32	0	0.0	32	100.0	
**CNS metastases**						0.26355
Yes	**10**	2	20.0	8	8	
No	**80**	7	8.8	73	73	
**Liver metastases**						0.52337
Yes	**51**	6	11.8	45	45	
No	**39**	3	7.7	36	36	
**hCG [mUI/mL]**						0.06762
<50,000	**46**	2	4.3	44	44	
≥50,000	**44**	7	15.9	37	37	
**AFP [μg/L]**						0.05653
<10,000	**66**	9	13.6	57	57	
≥10,000	**24**	0	0.0	24	24	
**Hemoglobin [g/L]**						**0.03884**
<100	**24**	5	20.8	19	19	
≥100	**66**	4	6.1	62	62	
**Dyspnea at rest**						**0.00001**
Yes	**24**	8	33.3	16	66.7	
No	**66**	1	1.5	65	98.5	
**Cough**						**0.02535**
Yes	**30**	6	20.0	24	80.0	
No	**60**	3	5.0	57	95.0	
**Chest pain**						0.33432
Yes	**11**	2	18.2	9	81.8	
No	**79**	7	8.9	72	91.1	
**Hemoptysis**						**0.00001**
Yes	**14**	6	42.9	8	57.1	
No	**76**	3	3.9	73	96.1	
**Weight loss ≥10%**						**0.00246**
Yes	**38**	9	23.7	29	76.3	
No	**52**	0	0.0	52	100.0	
**Pain grade ≥ 3**						0.51307
Yes	**22**	3	13.6	19	86.4	
No	**68**	6	8.8	62	91.2	
**Platelets [g/L]**						0.24624
≤300	**34**	5	14.7	29	85.3	
>300	**56**	4	7.1	52	92.9	
**Leukocytes [g/L]**						0.54373
≤10,000	**28**	2	7.1	26	92.9	
>10,000	**62**	7	11.3	55	88.7	
**CHT dose**						**0.00005**
Full	**63**	1	1.6	62	98.4	
Reduction	**27**	8	29.6	19	70.4	
**Induction CHT regimen**						**0.00001**
CBDCA	**1**	0	0.0	1	100.0	
CBDCA+VP-16	**1**	1	100.0	0	0.0	
EP	**25**	7	28.0	14	72.0	
BEP/T-BEP/VIP	**63**	1	1.6	66	98.4	
**DVT before CHT**						0.83622
Yes	**12**	1	8.3	11	91.7	
No	**78**	8	10.3	70	89.7	
**Primary prophylaxis of DVT**						0.08519
Yes	**36**	6	16.7	30	83.3	
No	**54**	3	5.6	51	94.4	
**Primary prophylaxis of FN**						**0.00157**
With (pegfilgrastim/filgrastim)	**45**	0	0.0	45	100.0	
Without	**45**	9	20.0	36	80.0	
**Bleeding grade ≥3**						**<0.0001**
Yes	**22**	8	36.4	14	63.6	
No	**68**	1	1.5	67	98.5	
**Mechanical ventilation**						**<0.0001**
Yes	**14**	9	64.3	5	35.7	
No	**76**	0	0.0	76	100.0	
**Septic shock**						**<0.0001**
Yes	**23**	8	34.8	15	65.2	
No	**67**	1	1.5	66	98.5	
**Febrile neutropenia**						**0.03976**
Yes	**39**	1	2.6	38	97.4	
No	**51**	8	15.7	43	84.3	
**FN despite primary prophylaxis**	***45***	*0*	0.0	*45*	100.0	–
Yes	**14**	0	0.0	14	100.0	
No	**31**	0	0.0	31	100.0	
**SII cutoff 844**						0.05653
< 844	**24**	0.0	0	24	100.0	
≥ 844	**66**	13.6	14	57	86.4	
**SII cutoff 1003**						0.19242
< 1 003	**26**	1	3.8	25	96.2	
≥ 1 003	**63**	8	12.7	55	87.3	
**SII cutoff 1842.46 (cohort median)**						0.29184
< 1 842.46	**45**	3	**6.7**	42	93.3	
≥ 1 842.46	**45**	6	13.3	39	86.7	
**NLR cutoff 3.3**						**0.03843**
< 3.3	**27**	0	0.0	27	100.0	
≥ 3.3	**63**	9	14.3	54	85.7	
**PLR cutoff 170**						0.3103
< 170	**34**	2	5.9	32	94.1	
≥ 170	**56**	7	12.5	49	87.5	

hCG, choriogonadotropin; AFP, alphafetoprotein; EC, embryonal carcinoma; YST, yolk sac tumor; NSGCT, non-seminomatous germ cell tumor; SII, systemic immune-inflammation index; NLR, neutrophil to lymphocyte ratio; PLR, platelet to lymphocyte ratio; CHT, chemotherapy; FN, febrile neutropenia; DVT, deep venous thrombosis; CBDCA, carboplatin; VP-16, etoposide; EP, etoposide cisplatin; BEP, bleomycin etoposide cisplatin; T-BEP, paclitaxel-BEP; VIP, etoposide ifosfamide cisplatin. Bolded texts highlight values with statistical significance P < 0.05.

Six patients had liver and two patients CNS metastases initially, as well. Five (56%) patients had RP LAP, while four (44%) patients had RP LAP ≥5 cm, and two (22%) of these had ≥10 cm. Patients with CS development had a higher mean level of hCG compared to patients without CS (661,977 vs. 258 368 mUI/ml, p = 0.017) and a lower mean AFP compared to patients without CS (462 vs. 17 903 μg/l, p = 0.01).

Predominant symptoms included dyspnea, hemoptysis, cough, and chest pain reflecting extensive metastatic lung involvement at the time of presentation. (**Table 2**). All patients with CS development had significant loss of weight initially. Patients with CS development had a lower mean level of total protein and a higher mean level of albumin compared to patients without CS (59.4 vs. 69.6 g/l, p = 0.004, and 32.0 vs. 26.1 g/l, p = 0.02, respectively). Patients with CS development had a higher mean level of leukocytes together with a higher mean level of NLR compared to patients without CS (16.25 10^9^/L vs. 12.47 10^9^/L, p = 0.04, and 9.9 vs. 6.0, p = 0.03, respectively). Patients with CS development had a lower mean level of hemoglobin compared to patients without CS (95.1 vs. 115.9 g/l, p = 0.005). Six patients had liver metastases, together with elevated AST, while four of them had an elevated level of total bilirubin, as well. Patients with CS development had a higher mean level of AST together with a higher mean level of total bilirubin compared to patients without CS (1.25 vs. 0.95 µkat/l, p = 0.047, and (23.1 vs. 17.6 µmol/l, p = 0.001, respectively).

None of the patients with CS could receive the primary prophylaxis of G-CSF due to the early CS development, while only one patient developed febrile neutropenia after the chemotherapy start. All nine patients needed ICU transfer after the chemotherapy administration, and all of them required mechanical ventilation as well. The median paO_2_ at the time of their admission to ICU was 53.6 (range: 38.2–76) mmHg, while seven patients had paO_2_ < 60 mmHg. All patients were febrile with an elevated level of C-reactive protein (CRP), procalcitonin (PCT), leukocytosis in eight patients, and leucopenia in the last one after the chemotherapy administration. Broad-range antibiotics and antimycotics were set up immediately. All patients required repeated bronchoscopic interventions due to respiratory tract bleeding; one patient had bleeding to the central nervous system (CNS) as well. Vasopressor support was needed in all of them while eight patients ended up in septic shock, and all developed ARDS, as well. At the day of the patient’s death, the white blood count was available for all patients with CS, with a median level of 21.5 g/l (range: 0.34–35.17), seven patients had leukocytosis, one patient had leucopenia grade 4, and in the last patient normal white blood count was present. All patients died due to multiorgan failure, and none of the patient was autopsied.

### Association Between the Patient Characteristics and CS Development

Univariate analysis revealed that factors associated with CS development were metastatic lung involvement ≥50% of lung parenchyma, choriocarcinoma elements in histology specimen, dyspnea, cough, hemoptysis, ECOG PS ≥2, weight loss, hemoglobin ≤100 g/l, and NLR ≥3.3 at the time of presentation (**Table 2**). In multivariate analysis, ECOG PS ≥2 and metastatic lung involvement ≥50% were independently associated with CS. All patients with these two characteristics developed CS compared to 0% with one or zero of these predictive factors (p < 0.000001) (**Table 3**).

**Table 3:** Multivariate analysis.

Variable	RR (95% CI)	p-value
ECOG 0–1 vs. 2–4	76.1 (6.2–933.5)	**0.00071**
Lung involvement ≥50% vs. <50%	120.1 (10.3–1397.6)	**0.00013**
Dyspnea at rest Present vs. absent	2.2 (0.2–21.0)	0.50481
HBG ≥100 vs. <100	0.7 (0.1–6.8)	0.77618
Weight loss Present vs. absent	9.9 (0.7–138.2)	0.08776
Hemoptysis Present vs. absent	0.6 (0.1–4.6)	0.65750

Bolded texts highlight values with statistical significance P < 0.05.

### Outcome and Survival

**Figure 2:**
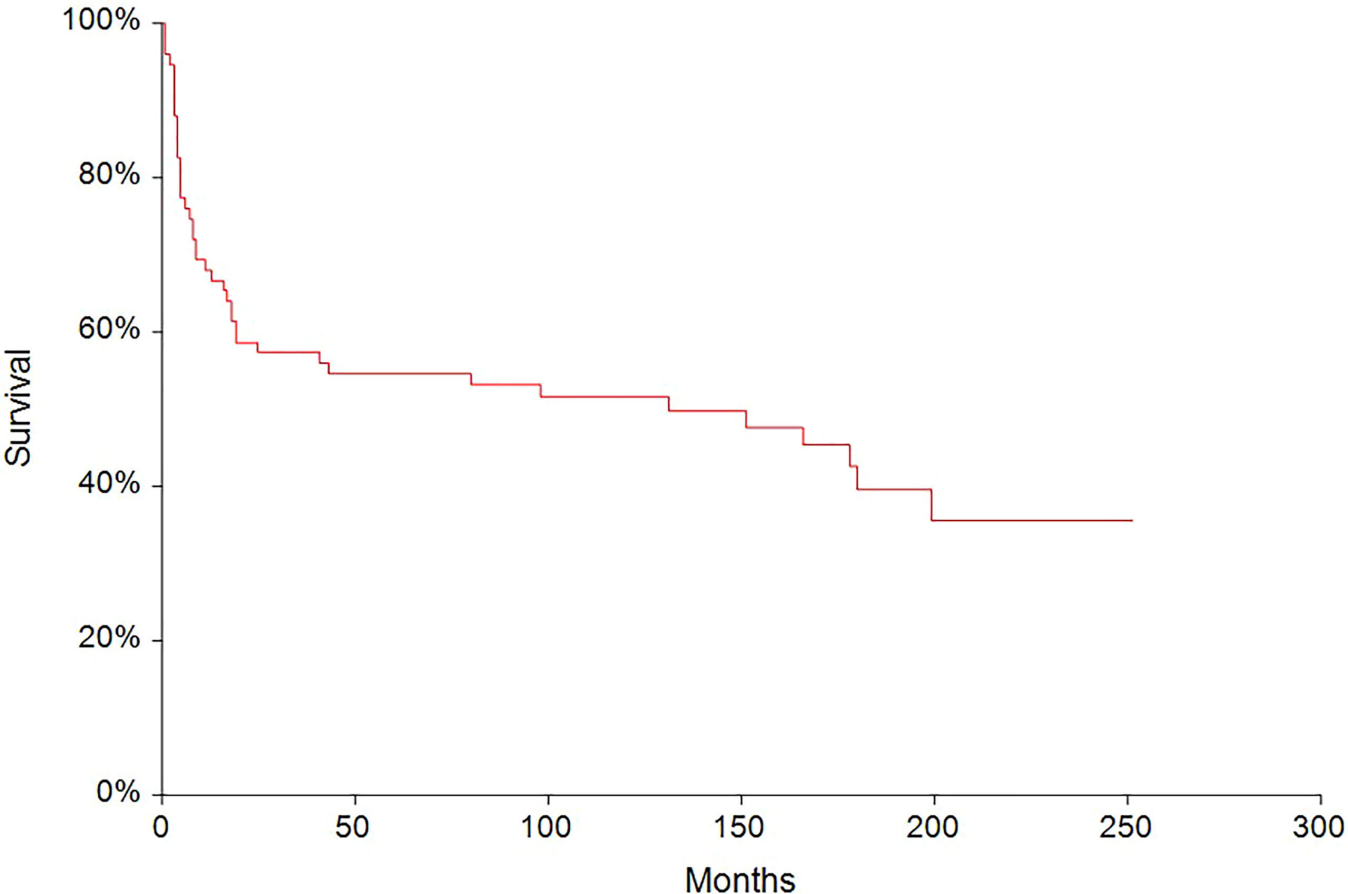
Kaplan–Meier estimates of probabilities of progression-free survival in all testicular germ cell tumor patients (n = 90), 5-year PFS = 54.6% (95% CI, 0.43–0.66).

**Figure 3:**
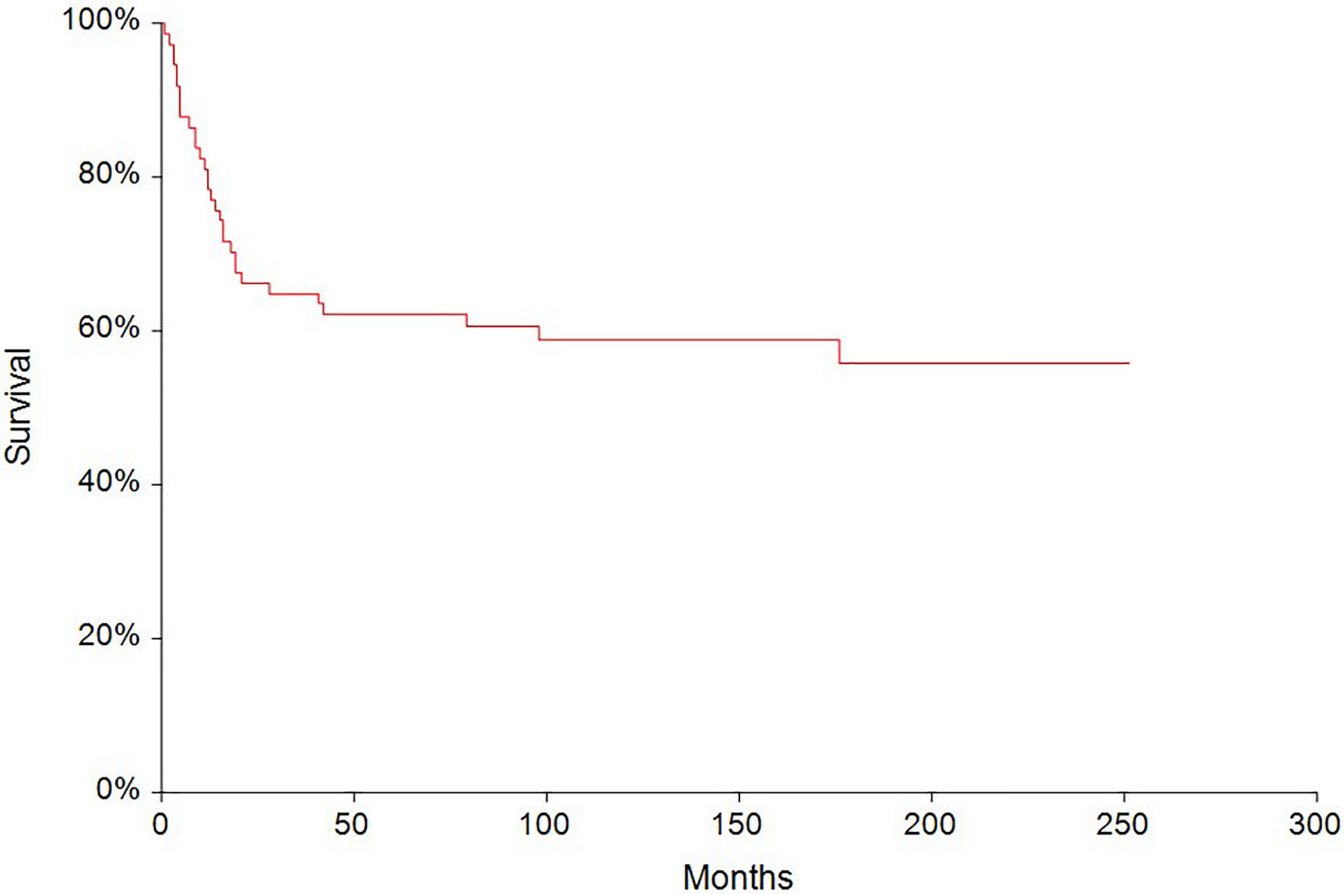
Kaplan–Meier estimates of probabilities of overall survival in all testicular germ cell tumor (N = 90), 5-year OS = 62.6% (95% CI, 0.51–0.73).

In the median follow-up of 45 months (range: 0–251 months), 56 (62.2%) patients experienced disease progression and 47 (52.2%) patients died. The 5-year PFS and OS were 54.6% (95% CI, 0.43–0.66) and 62.6% (95% CI, 0.51–0.73), respectively (**Figures 2**, **3**). All patients with choriocarcinoma syndrome development died shortly after the chemotherapy start with the median of 4 days (range: 3–35 days) due to consecutive ARDS development.

**Figure 4:**
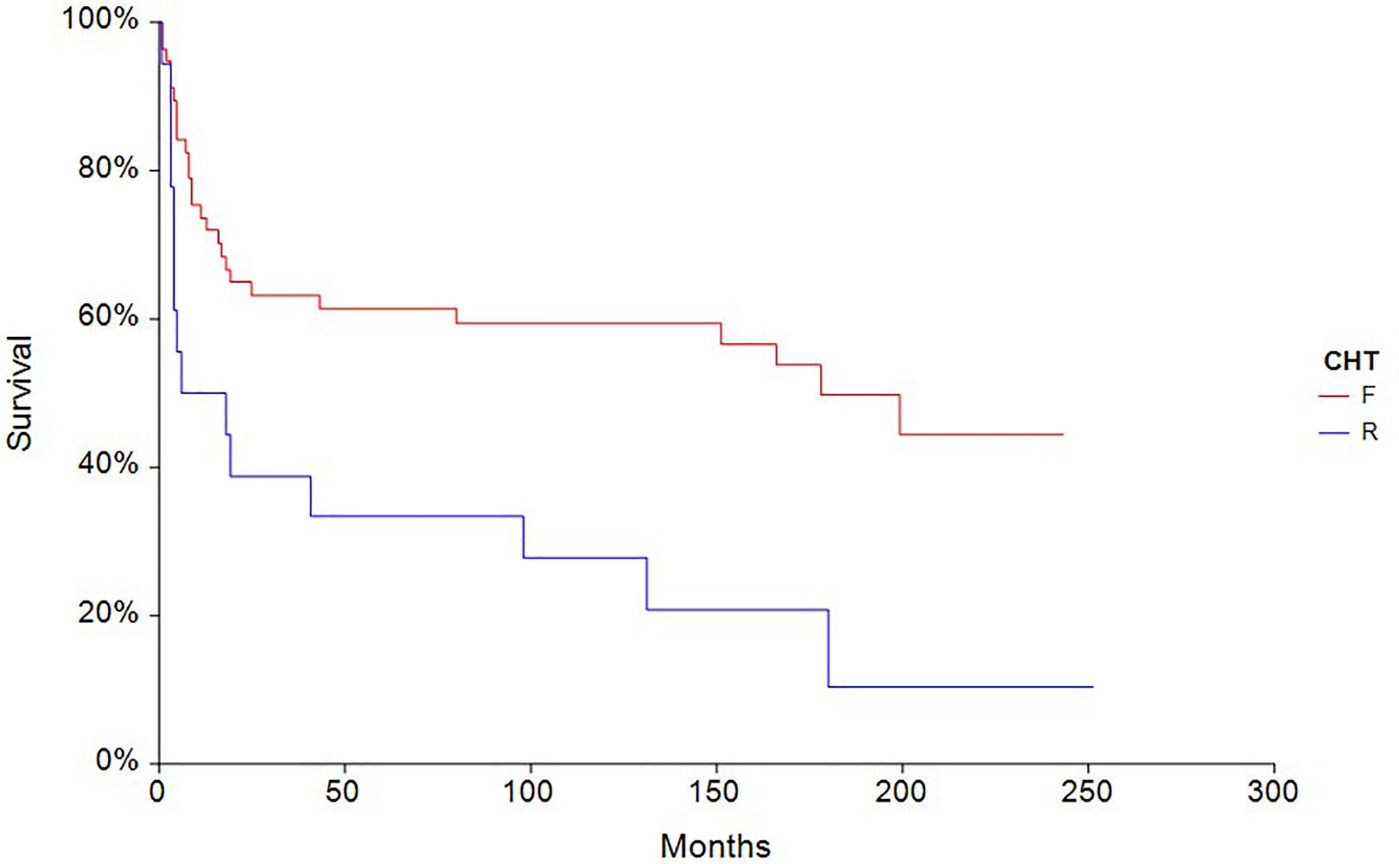
Kaplan–Meier estimates of probabilities of progression-free survival according to CHT regimen in testicular germ cell tumor patients (n = 90), (HR 0.40, 95% CI 0.18–0.88, p = 0.0027; F—full-dose CHT; R—reduced-dose CHT).

**Figure 5:**
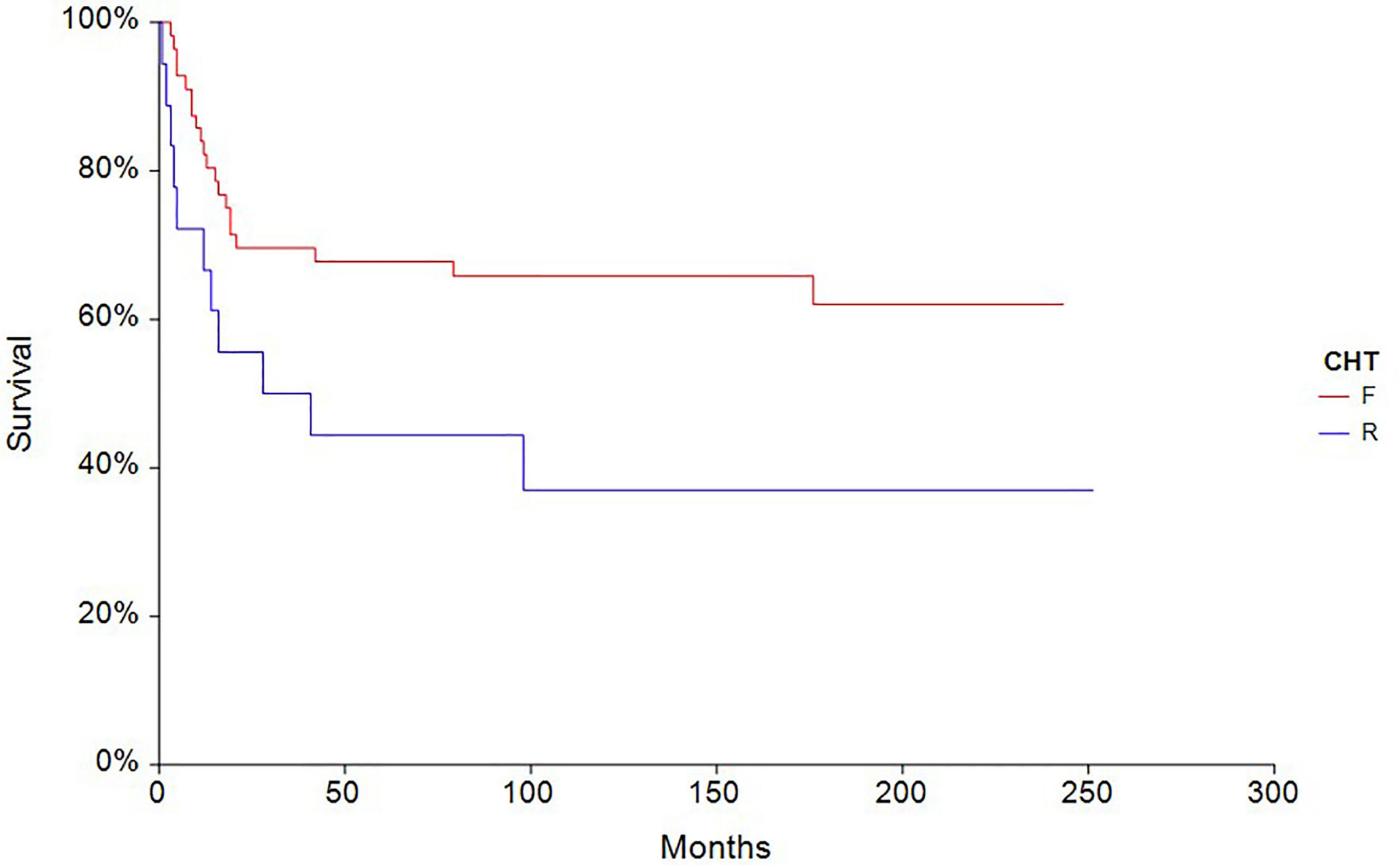
Kaplan – Meier estimates of probabilities of overall survival according to CHT regimen in testicular germ cell tumor patients (n = 90), (HR 0.46, 95% CI 0.19 – 1.10, p = 0.032; F — full-dose CHT; R — reduced-dose CHT).

The 5-year PFS in patients treated with full-dose chemotherapy (n = 63) was 61.4% compared to 33.3% (HR 0.40, 95% CI, 0.18–0.88, p = 0.0027) in patients treated with reduced-dose chemotherapy (n = 27) (**Figure 4**). Similarly, the 5-year OS rate in patients treated with full-dose chemotherapy (N = 63) was 67.9% compared to 44.4% (HR 0.46, 95% CI 0.19–1.10, p = 0.032) in patients treated with reduced-dose chemotherapy (N = 27) (**Figure 5**).

## Discussion

Management of patients susceptible to CS and consequent ARDS development within poor-risk GCTs remains a clinical challenge. Although these patients are potentially curable, there is a paucity of data evaluating the factors associated with CS development, the role of the intensity of induction chemotherapy on CS development, and the long-term outcome of those patients. To our knowledge, this is the first comprehensive retrospective analysis with the aim to search out the main risk factors of CS and consequent ARDS development at the time of presentation and after the chemotherapy administration. We observed that 10% of poor-risk patients developed ARDS in our study, which is lower compared to 19.6% in Moran-Ribon et al.’s study, who pointed out the possible selection bias by collecting those patients in their referral center (2).

Our data suggest that ECOG PS ≥2 with metastatic lung involvement ≥50% are the most powerful predictive factors of CS development. All patients with these risk factors developed CS and died shortly after chemotherapy administration. In addition, other factors identified in univariate analysis included presence of choriocarcinoma elements in histology specimen, dyspnea, cough, hemoptysis, weight loss, hemoglobin ≤100 g/l, and NLR ≥ 3.3 at the time of presentation.

Median time of ARDS development in our study was 1 day (1- 9 days), which is similar to Kirch et al. data with median of 2.5 days (0-14 days) (5). All our patients with CS died shortly after the start of chemotherapy with the median of 4 (3-35 days) due to consecutive ARDS development. This is shorter compared to previous studies with a median of 8 (4-130 days) and 12 days (6-35 days) respectively (2, 5).

In our group, all patients with ARDS development started the chemotherapy in our in-patient clinic with ICU transfer in the time of their respiratory worsening. This could be accounted for worse survival in our study, together with patient selection. All these patients had a very heavy tumor burden with mean 88.2% of metastatic lung involvement present, reflected by dyspnea and poor performance status. Unfortunately, initial paO2 at the time of patient’s presentation is missing in our records. This parameter reflects objectively the level of hypoxemia in patients with heavy tumor burden and it was the only predictor of intubation upon admission to ICU in Kirsch et al. study (5). We can only hypothesize, that treatment in ICU could possibly lead to better outcome in our patients, because management in ICU from the beginning of chemotherapy administration seems to be reasonable as all patients in mentioned studies were treated on ICU from the time of their presentation (2, 5, 6). Our univariate analysis revealed that dyspnea, hemoptysis and weight loss are the factors associated with ARDS development, which is in accordance with the initial presentation characteristics of super-poor-risk patients in Moran-Ribon et al. study (2). Choriocarcinoma elements were another significant risk factor defined, while hCG ≥50 000mIU/ml level was not, which is in accordance with Kirch et al. data, as well (5). It is unlikely that hCG may play a direct role in pathogenesis of CS syndrome, even though its development is more common in patients with high level of hCG, which reflects the tumor burden (5, 6). Only two patients with ARDS development had a hCG level <50 000 mIU/ml in our study.

All of them had NLR ≥ 3.3, majority of them leukocytosis and lowered level of total protein and albumin, at the time of their presentation. NLR is one of the many potential inflammatory prognostic biomarkers, which include easily measurable blood-based parameters such as CRP, leukocytes, platelets, LDH as well as reduction in protein and albumin (22, 23, 29–32). Elevated NLR, is a consistent poor prognostic factor across multiple cancer types and disease stages including TGCTs (33–38). Hypothetically, the systemic inflammatory processes already have been started in our patients, even before the chemotherapy administration. We did not find the significant association with SII a PLR, but there was trend toward it with SII, p = 0.057 (22). All our patients became febrile similarly to Kirch et al. and Moran-Ribon et al. patients and received empiric broad-range antibiotics. Despite that, all of them required vasopressor support due to evolved septic shock with the need of mechanical ventilation, as well. None of our patient could receive primary prophylaxis with G-CSF due to early ARDS development, while the expected neutropenia was noted in only one patient. Primary use of G-CSF is still recommended, which we perform in our tertiary center as well (39). Hypothetically, preemptive antibiotic treatment could be considered in individualized cases in patients with elevation of those biomarkers at the time of presentation due to positive results in mechanically ventilated patients (40–43).

Our univariate analysis showed that hemoptysis and lower hemoglobin were significant predictors of CS development, which could show the possibility of initiated CS even before the start of chemotherapy, hypothetically as well. Repeated bronchoscopies revealed acute hemorrhage into the respiratory tract in all our patients, which could aggravate respiratory failure after the chemotherapy administration. We suggest that those consecutive complications led to patient’s early death in our study. Poor performance status in patients with extensive lung metastases was the most powerful indicator of CS development in our study, which highlighted Tryakin et al. as the parameter in decision making for reduced induction chemotherapy in their study (13).

We believe that the risk of choriocarcinoma syndrome development cannot be fully prevented as all patients with massive lung involvement and poor PS developed CS in our study, despite the fact, that 89% of them were treated with reduced induction chemotherapy. Moran-Ribon et al. proposed that a standard dose of cisplatin-containing regimens should be recommended for super-poor-risk patients, because higher doses of cisplatin were connected to longer survival under mechanical ventilation compared to those with lower doses (6). This is in opposition to the Massard et al. approach with reduced induction chemotherapy, which lowers the rate of ARDS development and seems to prevent the risk of early death in their study (6). We agree with the Massard et al. approach, but on the other hand, selection of patients for reduced induction chemotherapy should be defined carefully, relying on adequate stratification system in the future, due to the possibility of long-term survival worsening with any modifications in this malignancy (33–38). Results from our study showed a shortened 5-year overall survival in patients with reduced induction chemotherapy, which can be a selection bias due to patient’s characteristics together with different treatment strategy (in-patient/ICU). The Massard et al. approach lowered the risk of ARDS and early death with reduced EP induction, but on the other hand, when we look at the data of long-term survivors, 80% of patients treated with full-dose chemotherapy, who did not die due to ARDS became long-term survivors, compared to 50% with reduced dose regimens (6), which is in accordance with our results. But we need to interpret these data very carefully due to very small patient numbers. Tryakin et al. concluded that dose-reduced first cycle of chemotherapy in patients with ultra-high tumor markers and/or poor performance status was associated with a significant decrease of life-threatening complications without worsening the overall survival. In their study, patients with reduced dose chemotherapy had significantly less hematological toxicity grade 4, but not infectious complications grade 3-4, including febrile neutropenia. Unfortunately, the data of overall survival in population with reduced induction chemotherapy were not presented (13).

Although the optimal therapy of this subgroup of poor-risk patients remains to be defined, the above mentioned factors associated with CS development based on our results together with biomarkers of lung damage, can create the risk- stratification model for reduced or full-dose chemotherapy in the future (44). Appropriate timing of continuing the full-dose chemotherapy in case of reduction needs to be defined, as well.

Our study has several limitations. The main limitation of our present analysis is the retrospective nature, with some important data missing in our records as well as the low number of patients with CS (n=9) and the fact that all of them died. Initial paO2 was measured in insufficient number of patients before chemotherapy initiation to perform analysis. Hypoxemic patients were immediately transferred to ICU, as clinically indicated. We can only hypothesize, that treatment in ICU could possibly lead to better outcome in our patients. It is possible that there was an accounted for selection bias, different induction regimens or different supportive strategies, which could modify the outcomes of the patients unrelated to mentioned invented predictive factors. All our poor-risk patients included in the study, had staging CT scan of thorax, abdomen and pelvis initially, however not all CT scans were digitally stored, so we could not perform digital volumetry. The CT scan data we had digitally stored would not be sufficient for our evaluation. Due to the fact, that 74% of our poor-risk patients had in our center initial X-ray as well, we could gain more reproductive date from X-ray evaluation in this setting. The X-ray is very important to do in this patient’s population susceptible to CS due to easy reproduceable changes in the lungs during treatment. It is easy to perform, and accessible. And due to this retrospective analysis, we can be confident, that this evaluation is sufficient for possible risk evaluation of CS development.

The number of patients with CS development from poor-risk patients should be interpreted carefully, since a selection bias by collecting those patients in our tertiary center, could be present. Even the very heavy tumor burden reflected by poor performance status, could account for their worse survival not necessarily the chemotherapy reduction or supportive care. There is no international consensus of CS definition, and therefore it is hard to compare the data across published studies. We suggest that the treatment approach in patients susceptible to CS development should not be concluded from our study and prospective studies are needed to validate the treatment approach in this patient’s population. From our point of view, it is important to perform international comprehensive study in this setting.

## Conclusions

In our retrospective analysis we described for the first time, ECOG PS ≥2 and metastatic lung involvement ≥50% as the most powerful factors associated with CS and consequent ARDS development in patients with poor-risk GCTs. Identified factors can create the risk-stratification model for these poor-risk GCTs patients. Due to its low prevalence, international collaboration and clinical trials utilizing new defined treatment strategies for patients susceptible to CS development are needed to decrease the mortality rate caused by this rare but highly fatal condition.

## Data Availability Statement

The original contributions presented in the study are included in the article/supplementary material. Further inquiries can be directed to the corresponding author.

## Ethics Statement

The studies involving human participants were reviewed and approved by the Ethical Committee of the National Cancer Institute of Bratislava, Slovakia. The patients/participants provided their written informed consent to participate in this study.

## Author Contributions

MM and KR participated in the conception and design of this study. MM and KR participated in data validation. VA, KK, MMa, NH, MC, VA, JO, PP, KR, ZS-M, JM and MM acquired, analyzed, and interpreted the data. KR and MM drafted the article, and all the authors reviewed it critically for its important intellectual content. All authors contributed to the article and approved the submitted version.

## Funding

This publication is the result of the implementation of projects no. APVV-20-0158, UICC-TF/19/643001 and 20190826/SVKNOI/1 NOI which was funded by the Scientific Grant Agency of the Ministry of Education.

## Conflict of Interest

The authors declare that the research was conducted in the absence of any commercial or financial relationships that could be construed as a potential conflict of interest.

## Publisher’s Note

All claims expressed in this article are solely those of the authors and do not necessarily represent those of their affiliated organizations, or those of the publisher, the editors and the reviewers. Any product that may be evaluated in this article, or claim that may be made by its manufacturer, is not guaranteed or endorsed by the publisher.
